# Boylston Medical Prize Questions

**Published:** 1860-11

**Authors:** 


BOLYSTON MEDICAL PRIZE QUESTIONS.—At a meeting of the Bolyston Medical Committee, held on the 1st of August, 1860, the annual prizes of ninety dollars each, or a gold medal of the same value, were awarded to Dr. John Bell, of New York, for the best essay on the question: "How far does the Microscope assist us in Surgical Diagnosis?"; and to Dr. David W. Cheever, of Boston, for his dissertation on "The Value and Fallacy of Statistics in the Observation of Disease."

For 1861 the following questions are proposed: 1. Excision of Joints. 2. Diagnosis and Treatment of Chronic Pleurisy. Essays on these subjects must be sent to Dr. Edward Reynolds, on or before the first Wednesday in April, 1861..

The following questions are proposed for 1862: 1. How far does the Microscope assist us in Medical Diagnosis? 2. On Nausea and Vomiting as Symptoms; under what circumstances do they occur, and what indications do they afford as to the seat and character of Disease? The dissertations are to be sent to the same direction, and at the same period above stated, 1862. The premium for the best dissertation on either of these subjects will be sixty dollars, or a medal of that value.

